# A Comparison of English and Dutch Long-Stay Patients in Forensic Psychiatric Care

**DOI:** 10.3389/fpsyt.2020.574247

**Published:** 2020-11-30

**Authors:** Dhanuja Senn, Erik Bulten, Jack Tomlin, Birgit Völlm

**Affiliations:** ^1^Division of Psychiatry and Applied Psychology, University of Nottingham, Nottingham, United Kingdom; ^2^Pompe Foundation, Nijmegen, Netherlands; ^3^Behavioural Science Institute, Radboud University, Nijmegen, Netherlands; ^4^Department of Forensic Psychiatry, University of Rostock, Rostock, Germany

**Keywords:** forensic mental health, length of stay, long-stay patients with mental illness, mentally disordered offenders (MDOS), service development

## Abstract

**Background:** A significant proportion of forensic patients in England are long-stayers. This can be problematic as individuals are kept in restrictive environments at potentially inappropriate levels of security for many years, sometimes decades. Improvements to the current English forensic mental health system to meet the needs of long-stay forensic patients more effectively might be informed by the Dutch service for long-stay forensic patients.

**Aims:** To compare the characteristics of representative samples of long-stay patients in England and in the Netherlands in an attempt to draw conclusions on the degree to which the Dutch service model might be relevant to England.

**Method:** This cross-sectional study explores the relevance of the Dutch service model by comparing the characteristics of representative samples of long-stay patients in England (*n* = 401) and the Netherlands (*n* = 102). Descriptive statistics and analyses of differences between groups are presented. The Risk-Need-Responsivity model was used to guide the selection of the study variables and structure the interpretation of the findings.

**Results:** Compared to their English counterparts, the long-stay Dutch patients were less likely to be diagnosed with schizophrenia, but more likely to have personality disorder and have committed sex offences. The English group were younger at first conviction and at first custodial sentence. The total number of offences and the proportion of violent offenders were similar, but the Dutch HCR-20 scores indicated a significantly higher risk of violence.

**Conclusions:** Whilst there may be barriers to adopting the Dutch service model in England, the differences in the characteristics of the two groups studied here do not necessarily preclude this approach.

## Introduction

In England, forensic care is provided at three levels of security (high, medium and low) with most facilities providing services at a single security level. High secure care is centralised at three hospitals whereas medium secure care is decentralised across 57 medium secure units. The current service model is based on a medical approach with emphasis on the mental health and security needs of the offender (1); inpatient treatment is focused on ameliorating symptoms, lowering risk and facilitating re-integration into society (2). The duration of such hospitalization is not fixed, however, and a significant number of patients remain in forensic care for lengthy periods. The length of stay in forensic psychiatric settings generally far exceeds that in general psychiatric units in the United Kingdom (UK) (3). A survey of 23 medium and all three high security hospitals found that on average 23.5% of patients were “long-stayers” (patients staying over 5 or 10 years in medium or high secure settings respectively) (4). In addition, there is evidence that for some patients the care provided has been at a level of security inappropriate to their needs and risk of recidivism. For example, Harty and colleagues (5) surveyed the responsible medical officers of 1,244 forensic patients in special hospitals (now called high security hospitals) and found that 40% were believed to be better placed in lower levels of security.

In the Netherlands, inpatient forensic care is provided predominantly by forensic hospitals which allow those with diminished criminal responsibility to be detained and treated in forensic psychiatric hospitals rather than in prison. Forensic care is provided at four different levels of security catering for different clinical and legal patient profiles (6). Some facilities may provide a single level of security while others will provide more than one. A judge may impose a forensic order (*Terbeschikkingstelling*; TBS), which provides care with the aim of protecting the public in addition to a prison sentence if a mentally disordered offender (MDO) is declared partially or fully unaccountable for a serious sexual or violent offence and there is judged to be a high risk of recidivism (7). Patients who receive a TBS order are judged to be at most risk of reoffending and are cared for in high security TBS hospitals (Forensisch Psychiatrisch Centrum; FPC). Other forensic or civil patients that pose a less significant risk of harm or reoffending are cared for in other secure settings (e.g., Forensisch Psychiatrische Kliniek, FPK; Forensische Verslavingskliniek, FVK; Forensisch Psychiatrische Afdeling, FPA).

Whereas the average length of stay in TBS facilities is about 8 years in 2017, the Dutch system has provided separate long-stay facilities since 1999. Aims of these long-stay facilities include providing psychiatric and medical care, optimising quality of life and encouraging the patient to accept their stay (8); a specific model of recovery is also used. The focus is thus on increasing autonomy and stabilization as opposed to reducing risk and encouraging re-entry into society for MDOs for whom such rehabilitation is not realistic within the short or medium term (9). Before a patient can be assigned to a long-stay service, they must have undergone treatment at two TBS hospitals for a total of at least 6 years, and treatment attempts at both institutions have not led to satisfactory clinical improvements in level of risk (10, 11).

Long stays in secure settings raise a number of issues. Forensic-psychiatric hospital services are expensive to run, and an extended hospital stay can give rise to ethical concerns. For example, detention at too high a level of security will be unnecessarily restrictive for the detained individual (12), whereas detainment at too low a level of security might pose serious risks to society. Furthermore, individuals may be held well-beyond the time they would have been incarcerated had they received a prison sentence as a non-mentally-disordered individual (13) which could violate basic human rights.

Arguments have been made that the current system of forensic mental healthcare in England should be improved so as to meet the needs of long-stay patients more effectively (14). There are currently no specific strategies for long-stay forensic care and no commissioned long-stay facilities in this country; long-stay patients remain distributed across the various high and medium secure settings. Given the lack of clarity regarding the best model of care for long-stay patients, it is appropriate to consider the applicability of any relevant service models for this patient group that have been adopted in other countries. The Netherlands is one of two European countries having dedicated services for long-stay forensic patients, the other being Germany. The Dutch long-stay services are reported to have helped prevent blockages in the TBS hospitals (9); they have also demonstrated reasonable levels of patient satisfaction (8) and quality of life (15). It has been suggested that UK policymakers might benefit from considering the Dutch experience in this field (16), but the degree to which the Dutch long-stay model would be relevant to the UK will depend in part on whether the two services cater for patients with similar needs and characteristics. A service model that has achieved some success in terms of patient satisfaction and cost effectiveness with one population may be less successful with another that is dissimilar. The research literature contains no direct comparison between English and Dutch forensic services that could inform on this issue, however. The current study seeks to address this deficit.

There are a number of practical differences between the English and Dutch systems. In terms of diagnosis, the Dutch system places no exclusions whereas in England patients with substance-related disorders alone cannot be detained under mental health legislation, potentially resulting in a different case mix between long-stay populations in the two countries. In terms of access, risk is a specific criterion for detention in the Netherlands whereas in England this is not the case. The criterion for admission most closely linked to this is that it is required that the “mental disorder from which the offender is suffering is of a nature or degree which makes it appropriate for him to be detained in a hospital for medical treatment” [§37 Mental Health Act, 1983 (as amended)]. Appropriate is an assessment made by the court with reference to the expert opinions of two register medical practitioners (usually forensic specialists, such as psychiatrists). This may result in a greater proportion of higher risk patients in the Dutch long-stay facilities.

In terms of service organisation, all TBS services are considered “high secure.” However, within TBS hospitals different wards provide some differentiation by security needs; as these are offered within one institution this potentially provides greater continuity as patients remain in contact with the same clinical team (17), whereas in England patients must transfer between hospitals as they move through the system and so will have contact with many different clinical teams (18). In terms of patient autonomy, the Dutch model appears less restrictive; for example, more patients are allowed to have supervised leave, to spend unsupervised time with family and to keep pets. In contrast, it has been suggested that the UK system is amongst the most restrictive in Europe with regards to sexual expression (19), and High Secure Service Directions impose significant restrictions on the amount of personal belongings allowed, on visiting rights and on procedures for searches (20).

The Risk-Need-Responsivity (RNR) model (21) has become widely used to guide forensic care both generally and in the two countries of interest here. The RNR model comprises three principles. The *Risk* principle stresses the importance of assessing the risk of re-offending and matching the intensity of treatment to the severity of that risk. The *Need* principle considers the importance of assessing criminogenic and mental health needs, and targeting these in treatment. The *Responsivity* principle stresses the importance of maximising the offender's ability to learn from or respond to a rehabilitative intervention by considering individual factors that might influence the intervention outcome.

One might hypothesise that the RNR model is not effective for long-stay forensic patients in either country. One possible explanation for this is that treatment is ineffective so that criminogenic or mental health needs do not diminish and the risk of recidivism does not decrease over time. It is also possible that these patients are not susceptible to treatment, which in turn relates to the Responsivity principle. In some cases, delayed discharge results from the absence of a suitable step-down facility to which the patient might be discharged, which can result in a poor match between treatment intensity and actual risk, besides having a negative impact on motivation. Invoking the principles of the RNR model in the comparison that is the focus of this study allows key variables (those relevant to Risk and Need) to be prioritised, regardless of the reasons these long-stay patients have failed to respond.

This cross-sectional study aims to compare the characteristics of representative samples of long-stay patients in England and in the Netherlands in an attempt to draw conclusions on the degree to which the Dutch service model might be relevant to England. Our main research question therefore was: are there any significant differences in the clinical characteristics, offending histories or current presentation between the samples in the two countries? If there were such differences, this might indicate that the two services cater for different patient groups and therefore the treatment models of one country (the Netherlands) might be of more limited relevance to that of the other (England).

## Materials and Methods

Data were obtained from two separate multicentre studies. The first (14) examined the characteristics and needs of long-stay patients in two high secure hospitals and 23 medium secure units in England and was funded by the National Institute for Health Research. The second [summarised in (22) and (23)] examined the characteristics of a representative sample of patients residing at two long-stay TBS hospitals in the Netherlands (FPC Pompestichting and FPC Veldzicht) and was supported by the facilities themselves.

The Netherlands as point of comparison was chosen for several reasons. First, the Dutch long-stay system has been explicitly developed to address the needs of a group of patients that are not benefitting from treatment as usual. This kind of service does not exist in the UK and it makes sense to investigate this further. Second, received more attention in the international forensic literature making it easier for readers to familiarize themselves with the system and draw their own conclusions about the applicability of this system in England and Wales. Third, other scholars have sought to draw parallels between these systems (16) and we hope to add to this literature. Fourth, this project was an ongoing collaboration with Dutch partners within the context of a European research network. Of course, the Dutch system is not a perfect system and in fact the Netherlands is often considered by some commentators be viewed uncritically through rose-tinted lenses and mythologized for liberal, progressive penal policy (24). However, for the reasons given above it is a sensible starting point to compare these two systems.

### Definition of “Long-Stay”

In previous UK studies, thresholds of 8 and 15 years have been used to differentiate long-stay patients in high secure samples, whereas for medium secure settings most studies have used a threshold of either 2 or 5 years (25). For the current study we defined long-stay in England as 5 years in medium secure care, or 10 years in high secure care, or 15 years in continuous secure care if patients had stayed in a combination of high and medium secure settings. This definition is in keeping with the 5-year threshold used in two previous medium secure studies (3, 26) and was informed by pilot data from one high secure care setting which suggested that 15% of patients stayed longer than 10 years. For the Netherlands, the definition of long-stay was effectively the admission criteria for the two participating TBS long-stay hospitals. This may be summarised as unsuccessful treatment at a minimum of two TBS facilities for a total of at least 6 years, where treatment success is defined in terms of a significant reduction in risk of re-offending.

### Selection of Patient Groups for Comparison

For the purpose of this study, we chose a two-way comparison between the Dutch sample and the combined English high- and medium-secure long-stay patients described above. We chose not to separate the two English sub-groups because:

1. Our UK and Dutch samples are similar in that both comprise some patients with high security and some with medium security needs. In the Dutch system, all TBS hospitals are “high secure” as described above and as such no long-stay services are provided at lower levels of security. Follow on services are low secure. Thus, the Dutch sample, although formally high risk, will contain a mix of patients with high and medium security needs, as is the case with our UK sample.
2. Some features of medium secure care in the UK parallel those of the high secure care of TBS patients in the Netherlands. For example, unsupervised leave is possible for patients in a Dutch high secure forensic facility (27), but not for a patient in a UK high secure hospital who would need to be in a medium secure setting for this to be considered. We argue therefore that it is important to include both high and medium UK care settings in our sample for any realistic comparison with the Dutch long-stay patients.
3. We have previously shown that there are in fact more similarities than differences between the high and medium secure groups even in our UK sample of long-stay patients who appear to constitute a unique group not easily defined by level of security (28).

Taking the above into account and the fact that the over-arching feature in our UK and Dutch samples is that they are long-stay patients, we argue that it is appropriate to consider them as two comparable groups which have significant length of stay as a core feature.

### Participants

Participants are summarised in Table 1. In England, participants were identified by examining the medical records for all patients resident on April 1, 2013. On this date, the total inpatient population within the three high secure hospitals and 57 medium secure units was 3,807 of which about 19% were estimated to be long-stay patients (14). The sample for this study was drawn from two high secure hospitals and 23 medium secure units. Selection of the medium secure units was on the basis of stratification by geographical region, sector (National Health Service or independent service provider) and unit size, with oversampling of units specialising in particular patient groups (women and patients with learning disabilities). A contact person was established at each unit to assist with the identification of those patients resident in these units who would meet the inclusion criteria on April 1, 2013. A total of 401 long-stay patients were identified of which 116 were resident in high secure settings and 285 in medium secure units.

**Table 1:** Study participants.

	**England**	**Netherlands**
**Long-stay forensic patients participating in the study**		
Total	401	102
In high secure care	116	
In medium secure care	285	
As percentage of forensic long-stay population	55.5%	68.9%
**Forensic population resident in 2013**		
Total	3807	1704^b^
In high secure care	715	
In medium secure care	3092	
**Long-stay forensic patients resident in 2013**		
Total	723^a^	148^c^
In high secure care	168	
In medium secure care	555^a^	
As percentage of forensic population	19.0%	8.7%

a *From Völlm et al. (16)*.

b *All TBS patients (29)*.

c *All long-stay TBS patients (29)*.

In the Netherlands, participants were randomly selected from within the two TBS long-stay hospitals, and as such were deemed to have long-stay status. In 2013, the total number of TBS inpatients in the Netherlands was 1,704 of which 148 were resident in the three long-stay TBS facilities (29). The sample for this study was drawn from two of the three long-stay TBS hospitals. A total of 102 long-stay patients were identified, comprising all patients in the smaller facility (*n* = 23) and a randomly selected sample from the larger second hospital^1^.

### Procedure

Detailed file reviews were carried out through examination of case records and medical notes. A comprehensive collection proforma was prepared, which covered all variables included in this study, and both English and Dutch data collectors were provided with instructions on administering the tool. In England, data were collected by unit staff and transmitted to the research team in fully anonymised form. In the Netherlands, data were collected by research students and recorded against a unique number to ensure that individual patients could not be identified. No new data were generated for this comparison study, the proforma was created for research purposes. Returned proformas were checked for inconsistencies and missing data by the research team and any queries were clarified with the data collectors.

### Variables and Measures

Where possible, we selected comparison variables which would fall within the three principles of the RNR model. From examination of patients' medical records, data were recorded in the following categories: sociodemographics, length of stay (as an indicator of responsivity), clinical diagnosis (the available patient notes, in both countries the DSM system is the most widely used to make as an indicator of mental health need; diagnoses were not generated for the purpose of this study and were taken from diagnoses), criminal history and the index offence that preceded admission (as indicators of criminological need), and risk assessment score (as an indicator of risk). Clinical diagnoses were as recorded by the psychiatrist responsible for each patient's care.

Assessment of risk was made using the last score before admission to the long-stay service in the Dutch sample and the one recorded in the last case review in the English sample on the Historical Clinical Risk Management-20 (HCR-20, version 2) instrument based on the in-patient context (30). This instrument, which has been reported as a reliable predictor of the risk of violent and non-violent offending (31, 32), is widely used in English forensic settings. In the Netherlands, risk assessment is mandatory and the HKT-R (*Historish, Klinish en Toekomst—Revisie*) is most commonly completed; however, the sites involved in our study used the HCR-20 which meant comparison was possible (33). At each site and in both countries, the HCR-20 version 2 was scored by a clinical psychologist who had undertaken training on how to use the HCR-20 provided by a trainer with experience in using and teaching others to use the tool. formal training in its use. Clinical psychologists in both countries require undergraduate and postgraduate training in psychology. Length of stay was calculated for the English patients from date of admission to continuous high or medium secure care, and for the Dutch patients from the date of admission to any TBS facility. Length of stay was calculated up to April 1, 2013 for both cohorts.

### Statistical Analysis

Quantitative data were analysed using Statistical Package for Social Sciences software (SPSS version 22; IBM Corporation, Armonk, NY, USA). Descriptive statistics were calculated for the English and Dutch samples separately. Categorical comparisons were made using cross-tabulation and chi-square tests with effect sizes computed as odds ratios (OR). For continuous data, means and standard deviations were determined; comparisons were made using *t*-tests and effect sizes estimated using Cohen's *d* (34) defined as the difference between two means divided by the pooled standard deviation. Values of *d* of 0.2, 0.5, and 0.8 are considered to represent small, medium and large effect sizes, respectively. Confidence Intervals (CI) were estimated at the 95% level. Mann–Whitney non-parametric tests were used to compare lengths of stay as this variable deviated from an approximately normal distribution. Two-tailed tests were used throughout. We considered a Bonferroni-corrected significance criterion of *p* < 0.005 to compensate for any Type I error inflation arising from multiple testing (35). Given 20 main exploratory tests, this would have entailed a Bonferroni-corrected alpha level of 0.0025 (i.e., 0.05/20). However, because the Bonferroni correction is widely regarded as conservative, we selected a significance criterion of *p* < 0.005 which was applied throughout.

### Ethical Considerations

For the English patients, the study was confined to data routinely collected by unit staff and transferred to the research team in a fully anonymised form. As such, it was deemed to constitute service evaluation by the sponsoring institution (Nottinghamshire Healthcare NHS Trust) since, according to guidance published by the UK National Research Ethics Service, studies classified as service evaluation and research that uses only routinely collected data made available to the researchers in anonymised form do not require ethical approval (36). Units were offered the option to exclude certain high-profile patients if they felt that data could not be provided in a way that would exclude incidental identification; one high secure unit excluded one patient under this procedure. The study was registered under Comprehensive Clinical Research Network Portfolio 129376, funded by the National Institute for Health Research and sponsored by Nottinghamshire Healthcare NHS Foundation Trust. For the Dutch patients, approval was granted by the ethics committee of the Pompe Foundation (the Netherlands). Privacy of the patients was assured by assigning a unique research number to each participant, allowing statistical analyses to be conducted on anonymous data collected from patient files.

## Results

Descriptive statistics and results of statistical comparisons are summarised in Tables 2–**6**. Effect sizes for those variables which differed significantly between the English and Dutch samples are described below.

**Table 2:** Sociodemographics of long-stay forensic patients^a^.

	**England (*N* = 401)**	**Netherlands (*N* = 102)**	***P*-value**	**Statistic**
Male gender, *n* (%)	344 (85.8)	100 (98.0)	** <0.001**	χ2 = 12.09
**Age, years**				
Mean (SD)	44.5 (11.3)	51.7 (8.9)	** <0.001**	*t*_(501)_ = 5.98
**Nationality**, ***n*** **(%)**				
British/Dutch	377 (94.0)	97 (95.1)	0.675	χ2 = 0.18
Other	24 (6.0)	5 (4.9)		
**Country of birth**, ***n*** **(%)**				
UK/Netherlands	364 (90.8)	70 (68.6)	** <0.001**	χ2 = 33.69
Other/unknown	37 (9.2)	32 (31.4)		
**Relationship status, *n* (%)^b^**				
Married or in a relationship	12 (3.1)	21 (21.6)	** <0.001**	χ2 = 41.73
Divorced/separated/widowed	44 (11.4)	12 (12.4)	0.796	χ2 = 0.07
Never married	329 (85.5)	64 (66.0)	** <0.001**	χ2 = 19.52
**Employment status pre-admission**, ***n*** **(%)^c^**				
Employed	86 (24.2)	6 (5.9)	** <0.001**	χ2 = 16.24
Unemployed/never worked	270 (75.8)	95 (94.1)		

a *Percentages may not add up to 100 because of rounding*.

b *Data missing from 16 English and 5 Dutch patients; total n = 482*.

c *Data missing from 45 English and 1 Dutch patient; total n = 457*.

*Values in bold are statistically significant at the level presented*.

### Sociodemographic Characteristics

The majority of patients in both countries were male, single, and unemployed at the time of their admission (Table 2). In comparison with the Dutch cohort, the English long-stay patients were less likely to be male, OR = 0.12; 95% CI (0.03, 0.50), *p* < 0.001, to be married or in a relationship at the time of offence, OR = 0.12; 95% CI (0.05, 0.25), *p* < 0.001, and they were more likely to have ever been employed, OR = 5.02; 95% CI (2.13, 11.92), *p* < 0.001. The Dutch patients were on average 7.2 years older than those in the English sample, *d* = 0.66; 95% CI (0.44, 0.88), *p* < 0.001. Although there was no significant difference between the two groups in terms of percentage of British nationals in the English sample and Dutch nationals in the Dutch sample, patients in the English sample were more likely to have been born in England than were patients in the Dutch sample to have been born in the Netherlands, OR = 4.50; 95% CI (2.63, 7.70), *p* < 0.001.

### Length of Stay

Lengths of stay are summarised in Table 3. A large proportion of patients in both cohorts had stayed within secure forensic facilities for at least 10 years (England 64.1%; Netherlands 96.1%). The Dutch long-stay patients had, on average, a longer length of stay [18.3 years vs. 14.6 years; *d* = 0.45; 95% CI (0.23, 0.67), *p* < 0.001]. The English sample were less likely to have stayed in three or more units since admission to their current spell of secure care, OR = 0.04; 95% CI (0.02, 0.10), *p* < 0.001.

**Table 3:** Length of stay of long-term forensic patients.

	**England (*N =* 401)**	**Netherlands (*N =* 102)**	***P*-value**	**Statistic**
**Length of stay, months**				
Mean (SD)	175.0 (103.9)	219.6 (76.8)	** <0.001**	*Z* = 5.984
**Length of stay category**, ***n*** **(%)**				
< =10 years	144 (35.9)	4 (3.9)	** <0.001**	χ^2^ = 40.07
>10–20 years	178 (44.4)	58 (56.9)	0.024	χ^2^ = 5.08
>20–30 years	53 (13.2)	34 (33.3)	** <0.001**	χ^2^ = 23.00
>30 years	26 (6.5)	6 (5.9)	0.824	χ^2^ = 0.05
Stayed in three or more units since first admission to current spell of secure care	165 (41.1)	96 (94.1)	** <0.001**	χ^2^ = 91.40

*Values in bold are statistically significant at the level presented*.

### Diagnosis

Schizophrenia was the most prevalent Axis I diagnosis in both cohorts (Table 4), although the English patients were more likely to have been diagnosed with schizophrenia, OR = 2.13; 95% CI (1.36, 3.32), *p* < 0.001, or schizoaffective disorder, OR = 7.61; 95% CI (1.82, 31.80), *p* = 0.001. Autistic spectrum disorder, OR = 0.09; 95% CI (0.04, 0.20), *p* < 0.001 and dementia, OR = 0.03; 95% CI (0.00, 0.24), *p* < 0.001, were less common in the English sample. Personality disorder was less common in the English sample compared to the Dutch sample, OR = 0.25; 95% CI (0.15, 0.42), *p* < 0.001. Although few patients presented with current symptoms of substance abuse, the English patients were less likely to have a history of substance dependence, OR = 0.43; 95% CI (0.28, 0.67), *p* < 0.001.

**Table 4:** Diagnosis of long-stay forensic patients.

	**England (*N =* 401)**	**Netherlands (*N =* 102)**	***P*-value**	**χ^**2**^**
**Axis I diagnosis**, ***n*** **(%)^a^**				
Schizophrenia Other psychotic disorder Schizoaffective disorder Bipolar disorder Depressive disorder Autistic spectrum disorder Dementia	232 (57.9) 12 (3.0) 53 (13.2) 13 (3.2) 23 (5.7) 10 (2.5) 1 (0.2)	40 (39.2) 11 (10.8) 2 (2.0) 7 (6.9) 8 (7.8) 22 (21.6) 8 (7.8)	** <0.001** ** <0.001** **0.001** 0.095 0.429 ** <0.001** ** <0.001**	11.38 11.31 10.58 2.79 0.63 49.67 26.68
**Substance dependence**				
Current diagnosis of SUD	33 (8.2)	6 (5.9)	0.457	0.55
Previous possible dependence	128 (31.9)	53 (52.0)	** <0.001**	14.18
**Axis II diagnosis**, ***n*** **(%)**				
Any personality disorder 2 or more personality disorders	186 (46.4) 73 (18.2)	79 (77.5) 24 (23.5)	** <0.001** 0.715	31.48 0.13

a *Definite and probable diagnoses grouped together*.

*Values in bold are statistically significant at the level presented*.

### Criminal History and Index Offence

The majority of patients in both groups had previous convictions for violent offences (England 79.6%; Netherlands 69.6%; Table 5). The Dutch long-stay patients were significantly older at first conviction (*d* = 0.36; 95% CI (0.12, 0.61), *p* = 0.004, and at first custodial sentence (*d* = 0.39; 95% CI (0.14, 0.63), *p* = 0.002, but the two groups were similar in terms of total number of offences. For the English sample their first offence was less likely to have been sexual than in the Dutch sample, OR = 0.33; 95% CI (0.18, 0.59), *p* < 0.001, and more likely to have involve property, OR = 15.31; 95% CI (3.71, 63.25), *p* < 0.001.

**Table 5:** Criminal history of long-stay forensic patients.

	**England (*N =* 401)**	**Netherlands (*N =* 102)**	***P*-value**	**Statistic**
**Total number of offences**				
Mean (SD)	15.3 (18.8) *n* = 395	12.6 (22.6) *n* = 94	0.230	*t*_(487)_ = 1.20
**Age at first custodial sentence (years)**				
Mean (SD)	21.3 (5.0) *n* = 222	23.8 (9.2) *n* = 90	**0.002**	*t*_(310)_ = 3.08
**Age at first conviction (years)**				
Mean (SD)	20.0 (8.2) *n* = 222	23.1 (9.3) *n* = 90	**0.004**	*t*_(310)_ = 2.91
**Previous convictions**, ***n*** **(%)**				
Violent Sexual Property Theft & Kindred Fraud & Kindred Police/courts/prison^a^ drugs Firearm/Offensive weapon Public order Other	319 (79.6) 110 (27.4) 228 (56.9) 209 (52.1) 44 (11.0) 126 (31.4) 50 (12.5) 96 (23.9) 125 (31.2) 93 (23.2)	71 (69.6) 50 (49.0) 13 (12.7) 56 (54.9) 7 (6.9) 4 (3.9) 6 (5.9) 21 (20.6) 8 (7.8) 33 (32.4)	0.032 ** <0.001** ** <0.001** 0.615 0.230 ** <0.001** 0.059 0.474 ** <0.001** 0.057	χ^2^ = 4.62 χ^2^ = 17.47 χ^2^ = 63.41 χ^2^ = 0.25 χ^2^ = 1.51 χ^2^ = 32.09 χ^2^ = 3.57 χ^2^ = 0.51 χ^2^ = 22.75 χ^2^ = 3.64
**Most severe sentence ever**, ***n*** **(%)**				
Indefinite imprisonment/life sentence Hospital order Prison 10+ years Prison 6–9 years Prison 4–5 years Prison 1–3 years Prison <1year Community service Other sentence Suspended sentence Total	36 (9.9) 276 (75.6) 5 (1.4) 10 (2.7) 3 (0.8) 11 (3.0) 4 (1.1) 3 (0.8) 16 (4.4) 1 (0.3) 365	1 (1.1)^b^ 19 (20.4) 4 (4.3) 6 (6.5) 7 (7.5) 39 (41.9) 17 (18.3) 0 0 0 93	0.006 ** <0.001** 0.070 0.082 ** <0.001** ** <0.001** ** <0.001** 0.380 0.040 0.613	χ^2^ = 7.71 χ^2^ = 98.47 χ^2^ = 3.31 χ^2^ = 3.03 χ^2^ = 15.60 χ^2^ = 115.5 χ^2^ = 50.02 χ^2^ = 0.77 χ^2^ = 4.22 χ^2^ = 0.26
**First conviction type**, ***n*** **(%)**				
Violent Sexual Property Theft & Kindred Fraud & kindred Police/courts/prison^a^ Drugs Firearm/offensive weapon Public order Other	130 (32.4) 33 (8.2) 94 (23.4) 128 (31.9) 5 (1.2) 7 (1.7) 10 (2.5) 16 (4.0) 15 (3.7) 28 (7.0)	39 (38.2) 22 (21.6) 2 (2.0) 31 (30.4) 1 (1.0) 0 2 (2.0) 2 (2.0) 4 (3.9) 14 (13.7)	0.267 ** <0.001** ** <0.001** 0.767 0.825 0.179 0.753 0.325 0.932 0.028	χ^2^ = 1.23 χ^2^ = 14.86 χ^2^ = 24.30 χ^2^ = 0.09 χ^2^ = 0.05 χ^2^ = 1.81 χ^2^ = 0.10 χ^2^ = 0.97 χ^2^ = 0.01 χ^2^ = 4.83
**Index offence type**, ***n*** **(%)^c^^,^^d^**				
Violent Sexual Property Theft & kindred Fraud & Kindred Police/courts/prison^a^ Firearm/offensive weapon Other	232 (69.5) 78 (23.4) 66 (19.8) 30 (9.0) 1 (0.3) 6 (1.8) 17 (5.1) 15 (4.5)	59 (57.8) 42 (41.2) 5 (4.9) 11 (10.8) 1 (1.0) 2 (2.0) 15 (14.7) 5 (4.9)	0.029 ** <0.001** ** <0.001** 0.585 0.373 0.914 ** <0.001** 0.862	4.75 12.44 12.65 0.30 0.79 0.01 10.62 0.03

a *This offence category refers to crimes committed against these organizations. For example, smuggling (conveying) prohibited articles into prison or perjury in judicial proceedings*.

b *This patient had, exceptionally, been sent by the Dutch Minister of Justice directly to a long-stay facility*.

^b^*Categories are not mutually exclusive so total percentage exceeds 100*.

c *For the English sample, percentage calculated in terms of those with an index offence*.

*Values in bold are statistically significant at the level presented*.

In both cohorts at least half the patients had an index offence that was categorised as violent (Table 5). The English long-stay patients were less likely to have an index offence that was sexual, OR = 0.44; 95% CI (0.27, 0.70), *p* < 0.001, or that involved a firearm or other offensive weapon, OR = 0.31; 95% CI (0.15, 0.65), *p* < 0.001, and were more likely to have an index offence that involved property, OR = 4.78; 95% CI (1.87, 12.21), *p* < 0.001. In terms of the sentence received for the index offence, the English patients were more likely to have been ordered to hospital, OR = 3.08; 95% CI (1.93, 4.92), *p* < 0.001, or have received indefinite imprisonment/life sentence, OR = 10.84; 95% CI (1.46, 80.27), *p* = .004, and were less likely to have received a prison sentence up to 3 years, OR = 0.07; 95% CI (0.03, 0.13), *p* < 0.001.

### Risk Data

HCR-20 risk assessment scores were available for 94% of the English sample and 97% of the Dutch sample (Table 6). The Dutch long-stay patients had, on average, higher HCR-20 risk management scores, *d* = 1.27; 95% CI (1.03, 1.51), *p* < 0.001, and higher HCR-20 total scores, *d* = 0.50; 95% CI (0.28, 0.72), *p* < 0.001.

**Table 6:** Risk assessment (most recent HCR-20 score) of long-stay forensic patients.

	**England (*N =* 377)^a^**	**Netherlands (*N =* 99)^b^**	***P*-value**	**statistic**
Total score mean (SD)	27.7 (5.5)	30.3 (3.9)	** <0.001**	*t*_(474)_ = 4.42
Historical score	15.6 (3.0)	15.3 (2.6)	0.364	*t*_(474)_ = 0.91
Clinical score	6.0 (2.6)	5.7 (2.0)	0.282	*t*_(479)_ = 1.08
Risk management score	6.0 (2.5)	9.1 (2.2)	** <0.001**	*t*_(474)_ = 11.26

a *Except for Clinical score where n = 380*.

b *Except for Clinical score where n = 101*.

*Values in bold are statistically significant at the level presented*.

## Discussion

This cross-sectional study compared the characteristics of patients in England and the Netherlands, who had experienced an extended stay in a forensic mental health setting, with the aim of informing on the degree to which the Dutch long-stay model might be relevant to the UK. The following Discussion has been structured according to the RNR principles in attempt to address the extent to which the two services cater for patients with similar needs and characteristics.

Detailed file reviews provided sociodemographic, clinical, offending and risk data for representative samples of long-stay forensic patients in each country. Just less than three-quarters of the 503 patients in the overall sample had a length of stay that exceeded 10 years and more than a fifth had stayed longer than 20 years.

The long-stay patients in both samples were predominately men with little experience of stable relationships or employment, which is suggestive of early disruptive lives as commonly found in studies of general forensic samples (37). Most were aged between 41 and 50 years. A psychotic disorder was the most common Axis I diagnosis among both cohorts. An Axis II (personality disorder) diagnosis was recorded for 46% of the English and 78% of the Dutch samples and is more prevalent than has been reported in studies of both UK and Dutch regular forensic psychiatric care (23, 38). One reason for this difference might be a difference in the systems of the two countries, not necessarily the patients themselves. The proportions of those with violent and sexual offences also appear to be higher than those reported in the general forensic population in these two countries (22, 39).

### Mental Health Needs

Those in the Dutch cohort were less likely to be diagnosed with schizophrenia but more likely to have received a clinical diagnosis of personality disorder, which indicates a significant difference in the balance of mental health needs between the two groups. This is likely to be a consequence of the difference between the English and Dutch systems. In the Netherlands, the choice between punishment and coercive measures is based on the assessed degree of responsibility of the defendant. When a court concludes that an offender had a degree of diminished responsibility instead of being fully responsible/not responsible, the judge may impose a prison term corresponding to the portion of psychological functioning that allowed the defendant freedom of choice alongside an order for forensic treatment. Assuming that findings of a complete lack of responsibility for a criminal offence are more likely for patients with a severe mental illness (e.g., psychosis) than a personality disorder, for which diminished responsibility might be more likely (40), this could imply a lower relative threshold for admission of patients with a PD into forensic services in the Netherlands than England.

The higher prevalence of personality disorder in the Dutch sample may also be viewed as a responsivity issue because forensic patients with personality disorder tend to be less responsive to pharmacological or psychological interventions (41), unlike many patients with psychosis. Patients with personality disorder are generally considered by clinicians as challenging and more difficult to manage (42); they tend to be viewed more judgementally than those with other mental disorders (43) and attract more negative attitudes from staff (44). These are likely to be important factors that would act to reduce responsivity.

In contrast, current substance dependence needs, regarded as an important dynamic factor in the RNR model (45), did not differ significantly between the two cohorts in as much as this can be implied from the current clinical diagnosis. On first inspection this appears counterintuitive. A patient whose only disorder is substance dependence cannot be detained under current English legislation, whereas the opposite is true in the Netherlands; an anticipated consequence is that substance dependence would be overrepresented in the Dutch cohort. In this study such overrepresentation was not found in terms of current diagnosis but was present for historical substance misuse. This may be because a greater number of patients in the Dutch cohort were admitted with substance use problems given the differences in legal admission criteria but that access to illicit substances is restricted in both settings making it hard for there to be current symptoms of substance use. This is especially likely given the lengthy durations of treatment, which would extend beyond detoxication periods. Alternatively, it might be the case that substance use is overlooked and insufficiently acknowledged in care.

### Criminogenic Needs

Some indication of criminogenic needs is provided by consideration of criminal history, and in this sense the needs of the two groups appeared matched through having a similar total number of offences and a similar high proportion of violent offenders. They differed in that the Dutch group contained almost twice as many individuals with a sexual index offence and were more likely to have an index offence that involved a weapon. It is difficult to draw conclusions about the relative severity of criminal history between the two groups, although the high prevalence of sex offenders together with the high rates of personality disorder in the Dutch sample might present particular challenges for staff in terms of problematic behaviours such as self-harm, causing staff and agencies to become polarised and poor treatment compliance (46).

In terms of sentencing for their index offence, more Dutch patients received a prison sentence up to 3 years, whereas more English patients had been ordered to hospital or received indefinite imprisonment or a life sentence. This is likely to be due in part to the different legal frameworks operating in the two countries. It may also arise because in the Netherlands most patients with a personality disorder receive a combined sentence of prison and TBS hospital, whereas patients with psychosis are more likely to be viewed as not accountable for their criminal offence and so are sent to a TBS hospital without a prison sentence.

Several other observed differences in treatment history may also be attributed to the disparate nature of the two forensic-psychiatric systems. First, the finding that patients in the Dutch sample were more likely to have received a prison sentence up to 3 years seems anomalous because an individual must have committed a severe crime that would warrant a prison sentence of at least 4 years to gain admission to the TBS system in the first place. However, whilst it is correct that the crime committed should in theory be severe enough to warrant a sentence of 4 years or more, in practice a Dutch court has considerable flexibility: at one extreme, it may choose to give no sentence at all, or at the other extreme choose to give a very long sentence. Second, those in the Dutch sample had experienced more changes in treatment settings compared to their English counterparts, which may arise in part from the requirement to have, in general, been treated in at least two different settings before being accepted into a long-stay facility.

### Responsivity

The Dutch patients had on average a longer stay in forensic care (by 3.75 years) and, perhaps as a consequence, tended to be older than those in the English group. This longer average length of stay may arise as a consequence of the proportion of Dutch forensic patient population that get admitted to long-stay facilities: if that proportion is small and relatively few are admitted, then the average length of stay is likely to be greater. No information was available that might indicate whether their longer stay was related to poor response to treatment or to systemic factors that can lead to the discharge process being blocked. The latter explanation seems unlikely, however, because the Dutch service has been reported as effective in preventing such blocking in the TBS hospitals (9), and also because concerns have long been expressed that a significant proportion of English patients remain at an inappropriate high level of security due to the absence of a suitable step-down facility (5, 47). Because each UK hospital has its own referral system, transfers between hospitals and step-down through security levels can be problematic, resulting in waiting lists and delays in treatment (16).

### Risk

The Dutch group had a significantly higher risk of violence as indicated by their HCR-20 scores, and particularly the HCR-20 risk management score which focuses on situational factors that can either aggravate or mitigate the risk of violence. The inevitable safety concerns that ensue may act to delay discharge and lead to a longer stay for the Dutch cohort. These longer stays could also arise because the Dutch long-stay service has a different philosophy from that of secure forensic services in the UK; a service designed specifically for long-stay patients which focuses primarily on quality of life and increased autonomy in a stable living environment may feel less pressure to discharge its patients even though there is pressure from the Dutch legal system, the Ministry of Justice and the independent advisory boards to prevent this. It is also possible that the focus on quality of life leads to a situation in which the patient is convinced that being in a long-stay facility is the least worst place to be; these patients may then themselves try to convince their lawyers, the court and the independent advisory boards to prolong their stay.

### Legal and Cultural Differences

Making international comparisons of criminal justice or healthcare systems is a valuable process. Doing so offers opportunities for learning from each other and sharing best practices (48). Particularly in a healthcare context, this might encourage a kind of race to the top where the best qualities or practices are taken from other systems and standards are harmonised. However, such comparative exercises are also fraught. Legal provisions, resource allocation, political priorities, cultures, concepts, languages and histories obfuscate the comparative process (49). This is certainly true for forensic services (50). A comprehensive comparison would take multiple facets of political, economic and cultural life into account and invest years in rich empirical research but could still suffer from linguistic or cultural misunderstanding. However, attempting such comparative work in spite of these limitations can be useful.

This study found differences between the two patient groups. It is likely that these differences were in some part due to the legal and cultural characteristics of the two jurisdictions as well as differences in demography or the psychopathological or criminological needs of the two countries [to see differences in rates of mental illness across the EU: (51)]. As already described, the legal criteria for admission into TBS services are different from forensic services in England and Wales. A further set of criteria are used to admit patients into long-stay care, which are not used in England and Wales. This tells us that a barrier to adopting such long-stay services in England and Wales might be the lack of legal or clinical criteria used to define a potential long-stay population.

Culturally there exist differences between the Netherlands and England and Wales that are relevant for these findings^2^. The Netherlands has long been popularly perceived as a liberal, progressive state—especially as regards penal policy [see for instance (52)]. The inclination of some to view the Dutch system in this way has been criticised as somewhat empirically detached, a consequence of a confirmation bias (24). However, studies do indicate that the Dutch penal and forensic systems are less punitive and restrictive than in England and Wales. Incarceration rates are much lower in the former: 61 per 100,000, compared to 140 in the latter (53). Dutch forensic patients are able to engage in sexual intimacy with other patients; this is not allowed in England and Wales (53). Patients in Dutch long-stay services are permitted leave into the community; this is only possible under very limited circumstances in high security settings in England and Wales (1).

Interviews with inmates and patients suggest these settings feel less punitive in the Netherlands. A study of inmates' in Dutch and UK prisons, many of whom had experienced prisons in both countries, reported that participants found prison staff in the Netherlands more friendly, responsive, humane, and fair, whilst being less formal, authoritarian, and disciplinary (54). Researchers in a separate study showed current and ex-forensic patients and carers in England a documentary about Dutch long-stay services (1). Analyzing participants' interpretations of the documentary, the authors found that when comparing services to those in England, (ex-)patients and carers felt these were less hierarchical, with less formal relationships with staff, and that patients had greater autonomy of movement in overall conditions of lower security, ultimately giving Dutch patients more responsibility, choice and privileges.

When considering these examples, it appears that Dutch long-stay services focusing on quality of life fit within a culture characterized by respect and autonomy instead of custody and punitiveness. This suggests that customs, beliefs, way of life, and the social organization of forensic care in England and Wales would have to change to become more accommodating of long-stay services in England and Wales.

### Limitations

The main limitation of this study lies in the nature of the two groups it seeks to compare. The Dutch long-stay patients are defined by their engagement with a purpose-designed long-stay service which has its specific admission criteria. On the other hand, the English sample is defined solely by the length of their stay in high and medium secure settings. Whilst the comparison reported here is valuable, it remains uncertain what the admission criteria might be for any new UK service designed specifically for long-stay patients. If those criteria were known and were used to define a subset of English long-stay patients that were more representative of those who would be cared for in a new or modified service, then their comparison with the Dutch sample might be more relevant. Those criteria are not known, and so this study remains limited by the possibility that the English sample on which it reports may not be fully representative of those long-stay patients who would be cared for in a new service.

As data in this study were extracted from patient notes recorded on hospital systems it is possible that there were incorrect imputations or inaccuracies by local staff. This may further have been complicated by the fact that research students in the Netherlands and unit staff in England completed the proforma for this study. This could affect the reliability of the data included in our study. However, we believe that our sample size is large enough to mitigate any possible inaccuracies and acknowledge that this is sometimes an unavoidable consequence of using hospital records. We do not believe the use of different groups (students and unit staff) will have substantially shaped the data included on the proforma as data were presented in patient notes without the need for interpretation. However, it is possible that unit staff might have filled out any missing data given their personal knowledge of the patients; having said that, the students were able to ask local staff for assistance, although we have no data on this. Future studies could consider, for example, conducting all HCR-20 or diagnostic assessments themselves.

It is a limitation that only two countries were included in this study. It would be a valuable exercise to investigate patient-level, as well as cultural, legal and systemic differences, across a wider number of countries. Some studies have attempted this (50), and whilst all acknowledge the difficulties of international comparison, call for more effort in this line of inquiry (55). A fruitful addition to such research would be the inclusion of a validated need assessment tool, such as the CANFOR or HoNOS-Secure (56). It is also a limitation that we did not compare patients based on a diagnosis of intellectual developmental disorder (ID). These data were not fully collected during fieldwork, and given their importance as a research topic, ID diagnoses should be included in future studies.

### Implications for Research

Further research is needed to compare the specific needs of these two cohorts of patients, preferably by conducting a formal assessment of needs of the two groups using the same measure. It would also be useful to explore HCR-20 risk scores and mental health needs scores at the item level to establish possible differences in specific needs. This would allow the samples to be broken down into subgroups on the basis of need. Follow up studies could be formulated to address the longer-term outcomes of the two groups. Of particular interest would be establishing what proportion Dutch patients stay in TBS long-stay facilities for a short period of time before returning to general TBS care, and what proportion stay in long-stay care without real prospects of transfer or discharge. Such needs-focused research should draw on qualitative research of carers' (57), staff (58), and patients' (59) perspectives and experiences of long-stay services.

### Implications for Practice

Although this study has not identified any differences between the Dutch and English populations of long-stay forensic patients that are so stark as to entirely preclude the adoption the Dutch approach in England and Wales *prima facie*, the study has found key differences between patients that would need to be factored into a larger investigation of the feasibility of providing long-stay services in a manner similar to the Netherlands. Other considerations pertain, as mentioned elsewhere in this article, to broader social and legal differences. These include differences between England and the Netherlands in the legal frameworks governing forensic psychiatric care, the fact that the UK service is based on a medical model focused on mental health need whereas the Dutch long-stay model focuses more on quality of life and patient autonomy, resistance to change by clinicians who place emphasis on treatment and recovery, and the UK environment in which regulatory bodies expect patients to receive treatment (14). Nevertheless, the large proportion of long-stay patients in England urgently requires a coordinated approach to deal with this patient group.

As has been suggested elsewhere (1) the institutional and cultural reticence to establish discrete long-stay facilities focusing on QoL are significant barriers. However, more realistic might be the development of long-stay services within pre-existing facilities. This could involve dedicating a single ward within a hospital to long-stay patients that meet explicitly outlined admission criteria. This setting could have different expectations about patients' progression through their pathways, provide patients with more autonomy and responsibility, shift the treatment focus from risk management to quality of life, and involve patients in the daily management of the ward. There should be a clear focus on relational security and use of de-escalation strategies. Patients should be offered the chance to engage in meaningful work for remuneration to structure and give extra purpose to their day. Patients should also be able to decorate their own rooms, possibly looking to the Netherlands in which there is the opportunity to keep plants and small pets. A focus on QoL would also involve greater opportunities for sexual intimacy with others inside and outside the hospital where appropriate. One challenge would be to avoid what one patient in high security services said of the possibility that such a high quality of life is achieved that patients lose motivation to ever leave long-stay services, that it would be “life sentence in disguise” [52: 117]. These efforts should be evaluated and the mental health and criminogenic needs of these patients compared to long-stay patients in the non-long-stay wards.

## Data Availability Statement

The data analyzed in this study is subject to the following licenses/restrictions: These data concern confidential patient information and will not be made publicly available. Requests to access these datasets should be directed to Birgit Völlm, birgit.voellm@med.uni-rostock.de.

## Ethics Statement

For the English patients, the study was deemed to constitute service evaluation by the sponsoring institution (Nottinghamshire Healthcare NHS Trust) and ethical approval was not required. For the Dutch patients, approval was granted by the ethics committee of the Pompe Foundation (the Netherlands).

## Author Contributions

DS: data acquisition, data entry, analysis and interpretation of data, and manuscript drafting and revision. EB: conception and design of the study, data acquisition, interpretation of data, and manuscript drafting and revision. JT: data interpretation and manuscript drafting and revision. BV: conception and design of the study, analysis and interpretation of data, and manuscript drafting and revision. All authors contributed to the article and approved the submitted version.

## Conflict of Interest

The authors declare that the research was conducted in the absence of any commercial or financial relationships that could be construed as a potential conflict of interest.

## Footnotes

^1^The Dutch sample includes one patient who was resident in a long-stay facility and had received a lifelong sentence; he had not, however, met the formal TBS qualification for long-stay status having been sent, exceptionally, approved by the Ministry of Justice, to a long-stay facility.

^2^Culture here is loosely defined, as by the Oxford Dictionary, as “the customs and beliefs, art, way of life, and social organization of a particular country or group.”

## References

[1] Edworthy E Majid S Vollm BA English vs Dutch high secure hospitals: service user perspectives. J Forensic Pract. (2018) 20:112–21. 10.1108/JFP-12-2016-0054

[2] Livingston JD Nijdam-Jones A Brink J. A tale of two cultures: examining patient-centered care in a forensic mental health hospital. J Forensic Psychiatry Psychol. (2012) 23:345–60. 10.1080/14789949.2012.668214 22815648 PMC3396355

[3] Sharma A Dunn W O'Toole C Kennedy GH. The virtual institution: cross-sectional length of stay in general adult and forensic psychiatry beds. Int J Ment Health Syst. (2015) 9:25. 10.1186/s13033-015-0017-7 26131018 PMC4485346

[4] Hare Duke L Furtado V Guo B Völlm BA. Long-stay in forensic-psychiatric care in the UK. Soc Psychiatry Psychiatr Epidemiol. (2018) 53:313–21. 10.1007/s00127-017-1473-y 29387921 PMC5842247

[5] Harty MA Shaw J Thomas SD Dolan M Davies L Thornicroft G. The security, clinical and social needs of patients in high security psychiatric hospitals in England. J Forensic Psychiatry Psychol. (2004) 15:208–21. 10.1080/1478994041000170396716582066

[6] Ministerie van Justitie en Veiligheid Informatieblad: Forensische zorg: Overzicht behandelinstellingen met verblijf. (2019). Available online at: https://www.forensischezorg.nl/introductie/keten-forensische-zorg/forensische-zorg-in-de-praktijk (accessed June 3, 2020).

[7] van Marle HJC The dutch entrustment act (TBS): its principles and innovations. Int J Forensic Ment Health. (2002) 1:83–92. 10.1080/14999013.2002.10471163

[8] Schel SHH Bouman YHA Bulten BH. Quality of life in long-term forensic psychiatric care: comparison of self-report and proxy assessments. Arch Psychiatr Nurs. (2015) 29:162–7. 10.1016/j.apnu.2015.01.004 26001715

[9] TBS TBS, vandaag over gisteren en morgen. Parlementair onderzoek TBS. [TBS, today, yesterday and tomorrow: An introduction to the parliamentary investigation of TBS]. Den Haag: SDU uitgevers (2006).

[10] Zwama M TBS-longstay: in historisch, hedendaags en toekomstig perspectief. [TBS-longstay: historical, contemporary and future perspectives]. Groningen: University of Groningen (2011).

[11] Sampson S Edworthy R Völlm B Bulten E Long-term forensic mental health services: an exploratory comparison of 18 European countries. Int J Forensic Mental Health. (2016) 15:1–19. 10.1080/14999013.2016.1221484

[12] Völlm B Bartlett P McDonald R Ethical issues of long-term forensic psychiatric care. Ethics Med Public Health. (2016) 2:36–44. 10.1016/j.jemep.2016.01.005

[13] Trebilcock J Weaver T Changing legal characteristics of dangerous and severe personality disorder (DSPD) patients and prisoners. J Forensic Psychiatry. (2012) 23:237–43. 10.1080/14789949.2012.668212n

[14] Völlm B Edworthy R Holley J Talbot E Majid S Duggan C A mixed-methods study exploring the characteristics and needs of long-stay patients in high and medium secure settings in England: implications for service organisation. Health Services and Delivery Res. (2017) 5:11 10.3310/hsdr05110 28263522

[15] Swinton M Carlisle J Oliver J. Quality of life for patients with a personality disorder comparison of patients in two settings: an English special hospital and a Dutch TBS clinic. Crim Behav Ment Health. (2001) 11:131–43. 10.1002/cbm.383 12048526

[16] de Boer J Gerrits J Learning from Holland: the TBS system. Psychiatry. (2007) 6:459–61. 10.1016/j.mppsy.2007.08.008

[17] McInerny T Dutch TBS forensic services: a personal view. Crim Behav Ment Health. (2000), 10:213–28. 10.1002/cbm.361

[18] de Boer J Whyte S Maden T Compulsory treatment of dangerous offenders with severe personality disorders: a comparison of the English DSPD and Dutch TBS systems. J Forensic Psychiatry Psychol. (2008) 19:148–63. 10.1080/14789940701830726

[19] Tiwana R McDonald S Völlm B. Policies on sexual expression in forensic psychiatric settings in different European countries. Int J Ment Health Syst. (2016) 10:5. 10.1186/s13033-016-0037-y 26848309 PMC4741020

[20] Department of Health The High Security Psychiatric Services (Arrangements for Safety and Security at Ashworth, Broadmoor and Rampton Hospitals) Directions 2011. London: Department of Health (2011).

[21] Bonta J Andrews DA The Psychology of Criminal Conduct. 6th ed. London: Routledge (2017).

[22] Nijman H Lammers S Vrinten M Bulten E (Too) long in tbs? A study on patients receiving forensic psychiatric tbs-treatment for 15 years or longer. Tijdschr Psychiatr. (2017) 59:9–19. 28098920

[23] Eckert M Schel S Kennedy HG Bulten E Patient characteristics related to length of stay in Dutch forensic psychiatric care. J Forensic Psychiatry Psychol. (2017) 286:863–80. 10.1080/14789949.2017.1332771

[24] Franke H Dutch tolerance: facts and fables. Br J Crim. (1990) 30:81–93. 10.1093/oxfordjournals.bjc.a047982

[25] Huband N Furtado V Schel S Eckert M Cheung N Bulten E Characteristics and needs of long-stay forensic psychiatric inpatients: a rapid review of the literature. Int J Forensic Ment Health. (2018) 17:45–60. 10.1080/14999013.2017.1405124

[26] Shah A Waldron G Boast N Coid JW Ullrich S Factors associated with length of admission at a medium secure forensic psychiatric unit. J Forensic Psychiatry Psychol. (2011). 22:496–512. 10.1080/14789949.2011.594902

[27] de Ruiter C Hildebrand M Risk assessment and treatment in Dutch forensic psychiatry. Neth J Psychol. (2007) 63:152–60. 10.1007/BF03061078

[28] Völlm B Edworthy R Huband N Talbot E Majid S Holley J. Characteristics and pathways of long-stay patients in high and medium secure settings in England; a secondary publication from a large mixed-methods study. Front Psychiatry. (2018) 9:140. 10.3389/fpsyt.2018.00140 29713294 PMC5911489

[29] Forensische zorg in getal Forensic Care in Numbers, 2010–2014. Custodial Institutions Agency, Ministerie van Veigheld en Justie, May 2015 Available online at: https://www.tbsnederland.nl/media/1067/forensische-zorg-in-getal-2010-2014-mei-2015-.pdf (accessed November 18, 2020).

[30] Webster C Douglas K Eaves D Hart S HCR-20: Assessing Risk for Violence (Version 2). Burnaby: Mental Health, Law and Policy Institute, Simon Fraser University (1997).

[31] Gray NS Taylor J Snowden RJ. Predicting violent reconvictions using the HCR−20. Br J Psychiatry. (2008) 192:384–87. 10.1192/bjp.bp.107.044065 18450665

[32] Douglas KS Reeves KA The HCR-20 violence risk assessment scheme: overview and review of the research. In: Otto RK, Douglas KS, editors. Handbook of Violence Risk Assessment. New York: Routledge/Taylor & Francis Group (2010).

[33] Bogaerts S Spreen M Ter Horst P Gerlsma C. Predictive validity of the HKT-R risk assessment for two and five-year recidivism in a cohort of Dutch forensic psychiatric patients. Int J Offender Ther Comp Criminol. (2018) 62:2259–70. 10.1177/0306624X17717128 28658999 PMC5960839

[34] Cohen J Statistical Power Analysis for the Behavioral Sciences. 2nd ed. Hillsdale: Lawrence Earlbaum Associates (1988).

[35] Dunn OJ Multiple comparison among means. J Am Stat Assoc. (1961) 56:52–64.

[36] National Patient Safety Agency Defining Research: NRES Guidance to Help Decide if a Project Requires Review by a Research Ethics Committee. London: National Research Ethics Service (2010).

[37] Macinnes M Macpherson G Austin J Schwannauer M. Examining the effect of childhood trauma on psychological distress, risk of violence and engagement, in forensic mental health. Psychiatry Res. (2016) 246:314–20. 10.1016/j.psychres.2016.09.054 27744234

[38] Doyle M Power LA Coid J Kallis C Ullrich S Shaw J Predicting post-discharge community violence in England and Wales using the HCR-20V3. Int J Forensic Ment Health. (2014) 13:140–7. 10.1080/14999013.2014.906517

[39] Ricketts D Carnell H Davies S Kaul A Duggan C First admissions to a regional secure unit over a 16-year period: changes in demographic and service characteristics. J Forensic Psychiatry. (2001) 12:78–89. 10.1080/09585180121913

[40] de Ruiter C Trestman RL Prevalence and treatment of personality disorders in Dutch forensic mental health services. J Am Acad Psychiatry Law. (2007) 35:92–7. 17389350

[41] Gibbon S Duggan C Stoffers JM Huband N Völlm B Ferriter M Psychological interventions for antisocial personality disorder. Cochrane Database Syst Rev. (2010) 16:CD007668 10.1002/14651858.CD007668.pub2 PMC4167848 20556783

[42] Newton-Howes G Tyrer P Weaver T Social functioning of patients with personality disorder in secondary care. Psychiatric Services. (2008) 59:1033–7. 10.1176/ps.2008.59.9.1033 18757597

[43] Markham D Attitudes towards patients with a diagnosis of ‘borderline personality disorder': social rejection and dangerousness. J Ment Health. (2003) 12:595–612. 10.1080/09638230310001627955

[44] Beryl R Völlm B. Attitudes to personality disorder of staff working in high-security and medium-security hospitals. Personal Mental Health. (2018) 12:25–37. 10.1002/pmh.1396 29024462

[45] Ogloff JRP Offender rehabilitation: from ‘what works' to what next? Aust Psychol. (2002) 37:242–52. 10.1080/00050060210001706936

[46] Lord A Perkins D Assessing and treating sexual offenders with mental disorders. J Forensic Practice. (2014) 16:94–109. 10.1108/JFP-02-2013-0012

[47] Thomas S Leese M Dolan M Harty M Shaw J Middleton H The individual needs of patients in high secure psychiatric hospitals in England. J Forensic Psychiatry Psychol. (2004) 15:222–43. 10.1080/14789940410001702283

[48] Goethals K Forensic Psychiatry and Psychology in Europe; A Cross-Border Study Guide. Forensic Psychiatry and Psychology in Europe. Cham: Springer International Publishing (2018).

[49] Nelken D Just Comparison. Comparative Criminal Justice: Making Sense of Difference Comparative Criminal Justice: Making Sense of Difference. London: SAGE Publications Ltd (2012). p. 25–39.

[50] Salize HJ Dreßing H. Placement and Treatment of Mentally Ill Offenders-Legislation and Practice in EU Member States. Lengerich Berlin Bremen Miami Riga Viernheim Wien Zagreb: Pabst Scientific Publishers (2005).

[51] European Commission The State of Mental Health in the European Union. Bruxelles: European Commission (2004).

[52] Downes D The case for going Dutch: the lessons of post-war penal policy. Political Q. (1992) 63:12–24. 10.1111/j.1467-923X.1992.tb00880.x

[53] Walmsley R World Prison Population List. 12th ed. London: International Centre for Prison Studies (2018).

[54] Dirkzwager AJE Kruttschnitt C Prisoners' perceptions of correctional officers' behavior in English and Dutch prisons. J Crim Justice. (2012) 40:404–12. 10.1016/j.jcrimjus.2012.06.004

[55] Tomlin J Lega I Braun P Kennedy HG Herrando VT Barroso R. Forensic mental health in Europe: some key figures. Soc Psychiatry Psychiatr Epidemiol. (2020) 1:3. 10.1007/s00127-020-01909-6 32651594 PMC7847441

[56] Keulen-de Vos M Schepers K Needs assessment in forensic patients: a review of instrument suites. Int J Forensic Ment Health. (2016) 15:1–18. 10.1080/14999013.2016.1152614

[57] Sampson S Foster S Majid S Völlm B Carers of long-stay patients' perspectives of secure forensic care: an exploratory qualitative study. Int J Forensic Ment Health. (2019) 18:305–15. 10.1080/14999013.2018.1552635

[58] Dutta S Majid S Vollm B S. D SM. Experiences and perceptions of nursing staff working with long-stay patients in a high secure psychiatric hospital setting. J Forensic Nurs. (2016) 12:111–9. 10.1097/JFN.0000000000000119 27533714

[59] Holley J Weaver T Völlm B. The experience of long stay in high and medium secure psychiatric hospitals in England: qualitative study of the patient perspective. Int J Ment Health Sys. (2020) 14:25. 10.1186/s13033-020-00358-7 32256688 PMC7104497

